# Book Reviews

**Published:** 1916-04

**Authors:** 


### Book Reviews.

Your Baby.—A Guide for Young Mothers. By Dr. E. B. Lowry, author of "Herself," "The Home Nurse," etc. Chicago, Forbes & Co., 1915. Price, $1.00.

Dr. Edith B. Lowry, the author of "Herself," "Confidences," "False Modesty," and a number of other books, has recently issued a new volume, entitled "Your Baby." The book is a splendid one and should be recommended by all physicians to young mothers and expectant mothers. The fact that 300,000 babies die every year in the United States is proof positive that the mothers should be educated in the care of their babies.

Dr. Lowry not only pleads for better babies, but plainly tells how to prepare for them; everything that is essential to the happiness and health of the mother and child is told. Nearly half the book is devoted to the mother's care of herself before the baby comes, and this part alone is invaluable to any expectant mother. A very timely chapter considers the various methods offered for painless childbirth, and much light is thrown on some fallacies and uncertain methods.

This book contains the latest and best approved methods for the care of the baby,—its feeding, clothing, exercise, sleep and training. It is full of common sense help and facts that many mothers might overlook. Like all Dr. Lowry's books it is permeated with an earnest spirit of helpfulness and wise, sane direction. If a prospective mother can have only one book on the subject of maternity and infancy it should be this safe and practical guide by Dr. Lowry, who is an authority of long experience in the health and care of women and children.

